# The Moral Authority of Consensus

**DOI:** 10.1093/jmp/jhac007

**Published:** 2022-06-25

**Authors:** Paul Walker, Terence Lovat

**Affiliations:** University of Newcastle, Callaghan, New South Wales, Australia; University of Newcastle, Callaghan, New South Wales, Australia

**Keywords:** *consensus*, *dialogic consensus*, *medical ethics*, *medical morality*, *moral authority*

## Abstract

Prompted by recent comments on the moral authority of dialogic consensus, we argue that consensus, specifically dialogic consensus, possesses a unique form of moral authority. Given our multicultural era and its plurality of values, we contend that traditional ethical frameworks or principles derived from them cannot be viewed substantively. Both philosophers and clinicians prioritize the need for a decision to be morally justifiable, and also for the decision to be action-guiding. We argue that, especially against the background of our pluralistic society, it is only via unforced dialogue and properly founded argumentation, aiming for consensus, that we can ascribe rightness or wrongness in a normative fashion to dilemmatic situations. We argue that both the process of dialogue, properly constituted, and the consensual outcome itself have moral authority vested within them. Finally, we argue that the consensual decision made is able to withstand moral scrutiny and is action-guiding, without claiming absolute moral authority in other contexts.

## I. INTRODUCTION

In his paper in this Journal, “Critical Reflections on Conventional Concepts and Beliefs in Bioethics,” J. Clint Parker (2019) comments on the role of dialogic consensus in modern bioethics. Specifically, “[m]edical ethics is full of disagreement. Agreement seems neither a sufficient nor a necessary condition for normative force” (Parker, 2019, 6). This followed upon his question “does consent born out of discourse always act as a sufficient condition for moral permissibility?” (Parker, 2019, 6). This question about whether consensus, specifically dialogic consensus, has moral authority or not is at the heart of this paper.

For some ethicists, the proposition, “consensus carries within itself moral authority,” is beset with difficulty because, from an ethical theory perspective, an argument can be put that certain ethical principles, rules, or frameworks are stand-alone and immutable, independent of context, and that we can make direct appeal to them in ethically challenging situations. These principles, rules, and frameworks draw their authority variously from religion, philosophy, natural law, harm-principles, human rights, and learned proclamations, among other sources.

We have previously identified concerns in medical contexts about making appeal solely to either deontology or teleology (Walker and Lovat, 2019). Truth-telling is a valued deontological precept which can nonetheless become troublesome in certain situations. Consider the situation wherein genetic testing has identified nonpaternity in a family of a highly traditional religious type. The consequences of truth-telling could be judged to be quite harmful, even extending in extreme cases to execution of the mother, abandonment of the daughter, and marriage ineligibility for the mother’s sister (Gray, 2015, 361). From a teleological perspective, funding mass immunization against rubella is likely to provide longer-term net benefit than increasing the number of neonatal intensive care beds to treat acute rubella. Nonetheless, if this were to be done, those infected with rubella who need acute care would have to be refused treatment.

Julian Savulescu (2015) has argued that medical ethics, to the extent that it locates its foundations in normative ethical theories, principles distilled from them, or statements of rules from various governing bodies, has failed. Amongst several examples, he includes misunderstanding the philosophical principles of autonomy and, hence, of coercion and consent, as a significant impediment to organ donation, medical research, distributive justice, and transplantation, among others. He argues in favor of an approach to morality which is “other-regarding.” This, he contends, is the philosophical concept which should underpin medical ethics, part of a wider position he takes that there should be greater attention to philosophy in medical ethics.

Johan Brӓnnmark notes that a common working assumption in normative ethics is that ethics is not domain-specific and that higher order moral principles can and should be appealed-to from within specific ethical domains. He describes a “kind of two-step, where we first ascend to the highly abstract level of one or a handful of completely general moral principles and then descend to the level of concrete domains where we can apply these principles” (Brannmark, 2019, 5). He argues that certain domains, including medical ethics, should not be grounded in general normative theories in this way.

To the extent that they are unexamined in their particular contexts, we argue that neither rules, traditional ethical frameworks, nor statements from august bodies can be substantive. Although ethical beliefs based on these might be (and typically are) strongly held, it is when they are applied without due regard for the context or situation in which the ethical decision is set, and without due consideration of the values of those on whom the decision will impact, that they become insufficient. Especially in our multicultural, multifaith communities with their diverse plurality of values, they do not carry the weight of moral authority without further substantial work. The “further substantial work” required follows from a process of inclusive, noncoercive, and self-reflective dialogue, among those whom the decision affects. That is, what is needed is a process of properly fortified argumentative dialogue that aims to reach a consensual decision amongst the participants.

We argue that properly constituted dialogue and consensus do contain, within themselves, a measure of moral authority. During the process of dialogic consensus (Walker and Lovat, 2016a), the participants on whom the decision impacts bring their own ethical values and principles to the process. Argumentation clarifies the bases for our ethical principles and holds them up to scrutiny in the specific context at hand. Thus, they might be found to be deficient in the extant situation, and a better decision for all might come to be seen as more acceptable to the participants. The decision made is action-guiding. We argue that such a process of dialogic consensus, along with its resultant decision, is capable of withstanding subsequent moral scrutiny.

## II. CONSENSUS

First, an important word about what we understand by the word “consensus” and what, in our understanding, is not properly termed “consensus.”

In the understanding, we posit here, *consensus* connotes general agreement, following argumentation, in reaching a decision about what is best for the group or the community making the decision. As such, consensus is necessarily tolerant of value pluralism.

Consensus is not unanimity, which denotes agreement by all participants, both publicly and privately. Nor does it denote acquiescence, which is agreement out of a sense of benevolence, of altruism, of coercion, or another reason that denies true argumentation. It does not imply a voting procedure or a simple majority decision, or “ethics-by-committee.” A recent clinical ethics paper reported that the clinical ethical decisions made in a hospital appear to have been based on an essentially unexamined, majority decision, made predominantly by clinicians, and based on the “settled morality . . . part of the fabric” of the hospital setting as articulated in hospital policies and guidelines that regulate interactions between clinicians and patients (Doran et al., 2015). We do not associate this methodology with moral authority. Similarly, we do not associate the past moral failures of an expedient male majority vote, such as condoning slavery, the refusal of citizenship for Indigenous people, or intransigence toward female suffrage, as consensus in any moral philosophical way. Nor is consensus simply *modus vivendi*.

We use the word consensus in a different, notionally stronger way, from that of Daniel Weinstock (2013, 2017). Weinstock defines consensus as “all parties agree that the position agreed upon is superior to the one they held at the outset, with respect to the issue at hand” (2017, 638). Weinstock uses the word “compromise” for the “position that, with respect to the issue at hand, is from the point of view of parties . . . in debate or negotiation inferior to the positions that both (or all) bring to a decision making process . . . but which both have reason to accept instead of the position they favour. They may favour X, when only the issue at hand is in view, but favour Y when all things are duly considered” (Weinstock, 2013, 539). Elsewhere, he describes compromise as agreement to “a course of action that we view as a sub-optimal response to some issue that requires a collective response, but with which we can live, and which we at any rate consider as better than the absence of an agreement” (Weinstock, 2017, 637). Kasper Raus et al. also use the word “compromise” similarly as “the process of resolving agreement through negotiation and normative concessions” (2018, 368).

These understandings of what is termed by these authors as “compromise” connote, in essence, our understanding of the word “consensus.” We prefer the word “consensus” because we associate the word “compromise” with agreement to an outcome reached by vote, or an outcome reached by trading-off some other agreement in exchange, or an altruistic or pragmatically made decision or conclusion reached so as to remain, for example, within a certain time frame, or simply so as to have the matter finalized. In a similar vein to our own view, Jane Braaten writes that “consensus, or agreement of opinion on the part of all concerned, is categorically distinct from compromise, or agreement by mutual concession” (1987, 347). Etymologically, “consensus” derives from the Latin *consentio*, “to feel together, to agree.” Seeking after consensus, in the sense that we understand it here, makes it possible for participants to accept a position which it is not reasonable for them to reject, in order to achieve a consensual decision. That is, it does not violate a threshold of acceptability or moral integrity relative to their own value set. Peter Caws agrees when he says “Consensus too may sometimes be reluctant, but . . . the members . . . will all agree that the outcome is, if not the very best in the opinion of each, at least thoroughly acceptable to each” (1991, 378). Finally, it may also be described as compromise *among* your principles, rather than *of* your principles (Bungo, 2013, 57). In fact, a region or range of reasonable decisions might prove to be acceptable in the circumstances at hand.

A consensual decision implies that it is action-inducing. When considering the place of minority beliefs in a wider society, Bhikhi Parekh has argued that the only way a society can decide which minority practices to allow is an “open-minded and morally serious dialogue with the minority spokesman and to act on the resultant consensus” (1996, 255). He recognizes that the outcome might not be ideal for all, but that it does allow for the showing of respect, deepening of mutual understanding, and arriving at a realistic and broadly acceptable decision.

We now examine consensus as a process and as an outcome. It is achievement of unforced consensual agreement, following argumentative dialogue, for which we claim moral authority.

### Consensus as a Process in Truth-Seeking

We begin by noting that from an epistemological perspective, Jürgen Habermas describes three “ways” of knowing (1972, 308). After collecting the facts (empirical-analytic knowing), we endeavor to understand the meaning of those facts (historical-hermeneutic knowing). It is reflection on the facts and consideration of their meaning which Habermas terms self-reflective knowing, and which is the most complete way of knowing. Searching out the truth in knowing is the basis for practical action. Michel Foucault also grounds the ethical self in what he terms self-scrutiny, rather than in principles, duties, consequences, or laws (Hugman, 2005, 109).

Habermas’ twin theories of discourse theory of morality (Habermas, 1993) and communicative action (Habermas, 1990) underlie consensus following dialogue. Habermas’ discourse theory of morality generalizes the Kantian categorical imperative, determined by ethical monologue, to a wider consensus-seeking dialogue. In communicative action, speech acts are orientated to understanding, and aim toward truth-seeking via participatory democracy. The use of language (linguistic or nonverbal communication) aims to reach a consensual decision in a dialogue in which all participants are “free to contribute and have equal opportunities to do so” (Scambler, 2001, 10; Scambler and Britten, 2001, 10); encapsulated as inclusive and noncoercive reflective dialogue.

Clinicians, patients, and their care-givers possess value systems that include frameworks of principles or ethics to which they can make appeal in challenging decision-making situations. It is appropriate that those who hold to a judgment about rightness or wrongness in decision-making situations can articulate the bases for their opinions about the situation. The majority of medical clinicians will likely be well-educated, financially secure, knowledgeable in their clinical area, and will often have well-established social networks, all of which inevitably influence their own value systems. Some patients need encouragement in a noncoercive dialogue in order to articulate their own values—those which matter most to them.

Especially helpful in clinical decision-making situations, in order to move from the ill-defined and nonspecific “best interests” of the patient, are the four Goods of the patient proposed by Edmund Pellegrino. These are the technomedical good, the perceptual good, the human good, and the *summum bonum* (Pellegrino, 1985). Elsewhere, we have argued for an approach to dialogue in clinical health care (Walker and Lovat, 2016b) (but usefully generalizable beyond that into the wider world of moral decision-making), which seeks to maximize the goods of the patient by finding the balance between a priori rules or imperatives, on the one hand, and empirical consequences, on the other hand. It borrows from phenomenological methodology by making the actual concrete situation of the patients in their real-world context the starting-point of the process. Finding this balance, or virtuous mean, can only be accomplished by having a proper conversation or dialogue.

Proffering the clinician’s value set as the only solution for patients and their family is inappropriately paternalistic. If, during the dialogue, clinicians impose their often predominantly deontological ethical norms on other members of their clinical group, an ethics committee or a case conference, then, as well as failing to respect the values and autonomy of the other participants, arguably the clinicians are using others as a means to their own end, as a means for executing their own moral vision. Especially in our postmodern era, our value-set cannot be uncritically binding on others who exist in their own context. For contemporary decision-making, we need another approach, one which exists in vivo at the bedside, set apart from the in vitro university ethical lecture hall. As such, it must successfully engage with the different cultural, religious, and familial imperatives that clinicians encounter among their patients.

Requirements of the dialogic consensus process include, in the ideal speech situation during a case conference or clinical ethics meeting: inclusivity of those who will be affected by the decision; that each participant mutually considers each other ready and willing to understand each other’s argument and value-claims in support of the moral contention; that all use language in the same way, including that technical terms are fully explained; that all allow the range of relevant arguments to be brought to the dialogue, seeking to understand each other’s values; that each can question an argument; and that there should be no overt or covert compulsion applied by or toward any participant in the argumentative discourse, which itself should be rational and impartial.

Given the importance of context, and the unique circumstances in every decision-making situation, we posit that, especially in our contemporary era, it is only via dialogue and properly founded argumentation under these specified conditions that we can normatively ascribe rightness or wrongness in dilemmatic situations. Meaningful engagement with the dissonant voices of our contemporary multicultural, multifaith society would seem to constitute a mature response to the problems of seeking a legitimate moral epistemology in our era. We say this because such a process makes proper allowance for circumstances, has room within itself for valid exceptions to imperatives and the weighting of different consequences, and aims to maximize the various goods of the patient and others on whom the decision impacts (consider the sexual partner of a man who risks erectile impotence following radical prostatectomy). As we have argued elsewhere, grounded in our intersubjectivity and interconnectedness, a process of dialogic consensus forces on us an active recognition of the viewpoints of others, regardless of how our own ethical values or life-choices differ from those of others.

Dissensus is not fatal to the process, and in fact is only possible in a situation of open and uncoerced dialogue. Moral discomfort during the dialogue is not fatal to the process either and, provided the arguments and values of the participants are understood, does not inevitably result in feelings of anger, guilt, powerlessness, or similar moral residue. However, aside from time constraints in real-world ethical consultations, there are further issues around the unique stake which the patient has in the decision; this is likely to engender significant emotions during the dialogue compared with the rarefactions of a thought experiment. As well, the staff in the dialogue need to be able to continue to work together, so the dialogue itself needs to be handled with sensitivity and compassion. That is, conflict resolution is a necessary part of real-world moral dilemma problem-solving, especially in clinical situations (Bungo, 2013, 30), in a way that classroom ethical deliberation does not require. The process of dialogic consensus is predicated on recognition of our essential intersubjectivity as human persons, and this, in our view, further fortifies its claim to moral authority.

Bruce Jennings highlights several notions important to our argument (1991, 457–8). First, he argues that the values holding society together must be grounded in a dialogical conception of rational consent. Etymologically, the word “consent” also derives from the Latin *consentio* (Caws, 1991, 377). Second, these values must be relatively neutral with regard to substantive conceptions of the good. That is, there is no Goodness Quotient which can be utilized to compare different goods as individuals perceive them. Third, the process which aims at moral consensus should favor deliberation that resolves moral conflict and disagreement, not simply one that brackets and sets such conflict aside. From a philosophical and a dialogic point of view, providing sufficient factual information, in a way that the patient can understand, is a significant contributor to patient autonomy. That is, if autonomy is to have real meaning, it must be founded on an understanding of the true facts of the situation. Furthermore, the process of consensus, to the extent that it is successful in fostering the conditions for the ideal speech situation, engenders respect for the patient and their values in the context of the medical illness at hand, and so further strengthens the patient’s autonomy in the situation.

Importantly, unlike ethical decision-making in the world at large, an ethically dilemmatic situation in clinical medicine is rarely one in which we are tempted to do wrong. Rather, it is a situation wherein we are trying to help the patient who is suffering an illness and are unsure which of our choices is most right. Those in the situation, on whom the decision impacts, are best placed to collectively determine what is best in their situation. Kasper Raus et al. suggest that the Habermasian process of “[d]eliberation and discussion is not what is needed to arrive at the right answer, but is, by contrast, what *makes the answer right*” (2018, 372). Raus et al. go on to say that “[w]hereas some ethical models see others as people who have to be convinced and brought to one’s own perspective, Habermas sees others as potential partners in a joint quest for the truth” (2018, 372). Meghan Bungo emphasizes that it “is not enough to *know* the correct answer; we need to *reach* the correct answer through a process based on the reasonable deliberation of the involved parties in order for it to be justified in the face of moral conflict” (2013, 81). Having the conditions for the ideal speech situation for argument and consensus is what provides the process with its normativity.

### Consensus as an Outcome in Truth-Seeking

We have contended that actual decision-making should be approached via engagement in the particular reality of people in their situation, set in their ethnic, religious and sociocultural backgrounds. Hence, it should be approached via moral dialogue—the second-person approach; in contra-distinction from first-person, agent-relative ethical monologue or third-person, agent-neutral prescription (Walker and Lovat, 2019, 78). Phenomenology recognizes the moral relationship in terms of an encounter, a meeting, a dialogue, an exchange, or a conversation, and so privileges the dialogical approach to morality (Verlinden, 2010, 94, 101). As noted, dialogic consensus requires an inclusive and noncoercive reflective dialogue in a situation where all participants have equal opportunities to contribute.

At the same time, the participants also have equal coresponsibilities to achieve a consensual decision. Thus, strategic action orientated to success, power, and aiming to influence is disavowed by the participants. Each participant considers each other to be accountable and willing to reach mutual understanding (Habermas, 2001, 147–8).

Bungo suggests that “reasonable deliberation,” which she characterizes as a “shared process of reason giving and openness to the reasons of others” (2013, 79), is required for a decision to be morally justifiable. She also emphasizes that the deliberation is other-regarding and requires that all participate in a conversation exploring the reasons and interests of the parties affected, with the desire to achieve resolution of the moral dilemma. Tempered quality of consideration is what, for Helen Longino, distinguishes legitimate from illegitimate consensus. It encompasses two considerations. First, all the participants have equal power in the dialogue. That is, “reasoning and argument be secured by unforced assent to the substantive and logical principles used in them,” rather than via strategic manipulation of the dialogue aiming to coerce (Longino, 2002, 131–2). Second, however, among the participants, is that the patient has a unique stake, and certain of the contributors should have the greater weight of their contributions recognized—for example, in prognosticating the outcome of a serious head injury, the contribution of an experienced neurosurgeon should be carefully listened to.

In thinking about assisted reproduction, Kurt Bayertz believes that “contemporary ethics is characterised by a vast diversity—or, if preferred, a chaos—of heterogeneous theories” (Bayertz, 1994, 1). In seeking moral consensus, like Habermas, Bayertz goes on to say that consensus has a claim to moral authority only when it is the result of communication aimed at intersubjective understanding. Properly, those involved are not concerned with bringing-about an agreement via strategic means, but via convincing them of the correctness of a normative statement using argumentations, “not merely factual consensus but *rationally founded* consensus” (Bayertz, 1994, 11).

Consider a situation where parents have conceived a child not of the sex they wanted, so they pose the question, “may we permissibly abort our healthy fetus because it is not the sex we wanted?” The question is whether the process of dialogue and a consensual decision seeks to find the truth or falsity in the situation that this fetus, these parents, relevant others, and the clinicians, are actually in; or whether the aim is to somehow “construct” truth, because there is a dearth of objective labels specifying truth or falsity, or an objectively validated, over-arching, context-independent truth or falsity conception (the ethical rule book) to tell us what is right and wrong. Herein, we are arguing in favor of the former. That is, we rely on a rigorous process of argument and counterargument predicated on understanding the values of the actual participants in order to find what is true or false for those in the dialogue, in this situation, at this time. We go so far as to suggest that none of the values that the participants hold at the start is objectively wrong; however, the participants do need to articulate their values in order to progress in the dialogue. Some participants may need to withdraw some way from their preferred ethical position in order to reach a consensual decision about what is right in the circumstances at hand. Alternatively, they may withdraw from the dialogue completely—although we believe that in our postmodern era, we have a responsibility to tolerance around conflicting values. We are not, however, generalizing the findings of this particular decision to the proposition, “aborting any healthy foetus because it is not the sex for which one hoped is wrong (or right).” That dialogue would involve different stakeholders or their representatives, but consensus would be equally binding on the community from which the representatives were drawn.

What if consensus is not achieved? Assuming the process of argumentation under the conditions specified has been followed, this means that the ethical values of one or several participants have trumped the ethical values of others in the dialogue. If this is the case, then the participants cannot be sure that the decision has moral authority. The significance of this prompts our belief that, notwithstanding practical difficulties in time-poor consultation spaces, there is a real need, in our postmodern era, to underpin decision-making with principles of awareness of intersubjectivity among us all, and tolerance toward the values held by other persons. These must be principles of conduct toward other people which apply no matter how our own ethical values, conceptions of the good, or life-choices differ. We borrow from Rainer Forst when we note that the dialogic process and the consensual decision must encompass “a degree of generality and a binding character that transcends the competing value conceptions” (2014, 63–4). By this, we mean that: first, the decision is generalizable in the sense of Habermas’ refinement of Kant—that it is accepted by all as agreed to by all; second, it is binding in the sense of being action-guiding for all the participants; and third, the process has taken into account the values held by the participants.

In the situation where a dialogue is held amongst, say, parents resistant to chemotherapy for their child who has an aggressive tumor, then an intelligent, frank, and open discussion of the values of the parents, the family, the clinicians, and the hospital or State authorities may still allow consensus to be reached. This is more likely if the clinicians can empathically listen to and understand the feelings and the fears of the parents about chemotherapy and the potential for their child to suffer, and the parents can understand that the clinicians seek to maximize the good of their child in a bad situation and are themselves aware of such things as unpredictable response rates to chemotherapy.

Consensus is much less likely if the parents’ beliefs are faith-based around not interfering with the will of God. This is a difficult issue because a steadfastly held intractable belief, which is based exclusively on revealed truth, and hence, cannot be rationally or logically proven or disproven, is outside the domain of rational argumentation. Thus, while a serious and sensitive attempt should be made by the participants to come to understand and resolve the disjunction of beliefs, if in effect participants have closed their ears and minds to any argument at all and will not engage in a rational dialogue, then it can be argued that they have excluded themselves from the dialogic decision-making process, and the State, for example, may adopt a proxy role seeking to maximize the child’s good in the parents’ stead. A similar issue arises with those who have delusional thought processes or are intellectually unable to comprehend the situation and the arguments. In these latter two instances, a proxy decision-maker can substitute. While in the strongly held religious belief context, the parents may refrain from making a decision themselves because it would be in contravention of all they believe in, that which gives meaning to their own lives, but will allow the group to make the decision, and then act on that decision by not attempting to prevent chemotherapy from going ahead. Ideally, because the dialogue was held with the parents in a respectful way, they may feel engaged in the process and continue to care for their child after chemotherapy is commenced, rather than abandon the child.

We now consider whether a consensual outcome can withstand moral scrutiny.

## III. WITHSTANDING MORAL SCRUTINY

Arguably, philosophers are primarily concerned with the conditions under which a decision is able to be morally justified. Clearly, clinicians also prioritize this concern in their dealings with patients, but, importantly, they are also concerned with compliance with the consensual decision in binding moral agents to participate in actions and to provide assurance that the participants are doing the right thing. Put another way, the decision reached after the process of dialogic consensus needs an accompanying sense of “oughtness” or “shouldness” about it. This is completely different from what is decided after what might be termed “ethics-by-committee” or “group think” or a process of voting initiated by the senior clinician.

In considering the question as to whether the decision made following a process of dialogic consensus can withstand moral scrutiny, the first point to consider is just whose moral scrutiny the consensual decision must withstand. If only those on whom the decision impacts, and who were therefore part of the dialogue, then the question becomes moot because the decision made after argumentation was consensual. In other words, the best-in-the-actual-circumstances decision was made. As we have iterated, dialogic consensus does not claim absolute moral truth in all circumstances, but merely that consensus after dialogue is the best truth in the particular situation.

The next question is whether those providing the moral scrutiny have a time limit on that scrutiny. Clinical decisions typically have a definite time frame within which to make the decision. Making no decision is making a decision, and nature may take its course. Academic commentators on ethical dilemmas rarely limit themselves with a time frame for the decision (think of thought experiments). Importantly, too, for how long after the decision is made must it be able to withstand moral scrutiny? Must it still be defendable a century later (Bungo, 2013, 28)? We think not. While welcoming academic analysis of the decision made in an individual clinical case, as inevitable and necessary for the benefit of future decision making in clinical case conferences, pragmatically, the decision reached is uncommonly reversible (albeit subject to ongoing review when the clinical condition changes). More important for helpful critique is whether the principles of a noncoercive dialogue were followed, with due attention to ideal speech considerations. Hence, the facilitator of a case conference or clinical ethics meeting plays a vital role.

A similar point to make clear is that moral scrutiny is likely, in practice, to be displaced temporally, possibly well after the decision has been put into effect. Once again, we should be clear that the process of dialogic consensus is not seeking absolute moral truth for all time in all situations. Rather, it seeks to make the best decision that this group of human beings can make, in the particular circumstances, at the time.

## IV. CONCLUSION

The bedside consultation, clinical ethics consultation, or case conference around ethically dilemmatic situations value the process of and encourage participants toward achieving an unforced consensus among the stakeholders of clinician, patient, and relevant others after a process of dialogue. When undertaken properly, the conversation we have aims to engage with the actual beliefs and values of the participants. Thus, decision-making about rightness or wrongness in the situation at hand is relocated away from being merely a monological reflection on imperatives, utility, or an *agapeic* calculus, into a social space cognizant of the other, where these ethical values are brought into the light, via having a conversation, discourse, or dialogue. We argue that such a process—of placing the decision to be made into its context and giving adequate consideration to the ethical values of those on whom the decision will impact—is more likely than not to achieve the morally correct decision in the particular situation under consideration, without laying claim to absolute moral authority in all circumstances. It is for these reasons that we propose that properly formed consensus has moral authority vested in it.

We do not disagree that the contention that consensus has moral authority demands further analysis. However, it seems more likely to be an arguable contention in clinical encounters where clinicians, patients, and their families dialogue about how best to maximize the good of the patient who is suffering. That being said, we also contend that as a response to the disparate values and conceptions of the Good in our contemporary postmodern era, dialogic consensus can also be applied broadly beyond bioethics to address wider societal issues with potentially similar levels of moral authority.

## References

[CIT0001] Bayertz, K . 1994. The Concept of Moral Consensus: The Case of Technological Interventions in Human Reproduction. Dordrecht, Netherlands: Springer.

[CIT0002] Braaten, J . 1987. Rational consensual procedure: Argumentation or weighted averaging? Synthese 71(3):347–54.

[CIT0003] Brannmark, J . 2019. The independence of medical ethics. Medicine, Health Care, and Philosophy 22(1):5–15. 29770916 10.1007/s11019-018-9842-1 PMC6394499

[CIT0004] Bungo, M. E . 2013. Searching for consensus: Shared decision making and clinical ethics. PhD Diss. Knoxville, TN: University of Tennessee.

[CIT0005] Caws, P . 1991. Committees and consensus. The Journal of Medicine and Philosophy 16(4):375–91. 1895023 10.1093/jmp/16.4.375

[CIT0006] Doran, E., J. Fleming, C. Jordens, C. L. Stewart, J. Letts, and I. H. Kerridge. 2015. Part of the fabric and mostly right: An ethnography of ethics in clinical practice. Medical Journal of Australia 202(11):568–90. 26068692 10.5694/mja14.00208

[CIT0007] Forst, R . 2014. Ethics and Morals. New York: Columbia University Press.

[CIT0008] Gray, B . 2015. Culturally competent bioethics: Analysis of a case study. Journal of Bioethical Inquiry 12(2):361–7. 26016616 10.1007/s11673-015-9636-6

[CIT0009] Habermas, J . 1972. Knowledge and Human Interests. London, United Kingdom: Heinemann Educational.

[CIT0010] ———. 1990. Moral Consciousness and Communicative Action. Cambridge, United Kingdom: Polity Press.

[CIT0011] ———. 1993. Justification and Application: Remarks on Discourse Ethics. Cambridge, United Kingdom: Polity Press.

[CIT0012] ———. 2001. On the Pragmatics of Social Interaction: Preliminary Studies in the Theory of Communicative Action. Cambridge, MA: MIT Press.

[CIT0013] Hugman, R . 2005. New Approaches in Ethics for the Caring Professions. Hampshire, United Kingdom: Palgrave MacMillan.

[CIT0014] Jennings, B . 1991. Possibilities of consensus: Towards democratic moral discourse. The Journal of Medicine and Philosophy 16(4):447–63. 1895027 10.1093/jmp/16.4.447

[CIT0015] Longino, H. E . 2002. The Fate of Knowledge. New Jersey: Princeton University Press.

[CIT0016] Parekh, B . 1996. Minority practices and principles of toleration. The International Migration Review 30(1):251–84.

[CIT0017] Parker, J. C . 2019. Critical reflections on conventional concepts and beliefs in bioethics. The Journal of Medicine and Philosophy 44(1):1–9.

[CIT0018] Pellegrino, E . 1985. Moral choice, the good of the patient, and the patient’s good. In Ethics and Critical Care Medicine, eds. J. C. Moskop and L. M. Kopelman, 117–38. Dordrecht, Netherlands: D. Reidel Publishing.

[CIT0019] Raus, K., E. Mortier, and K. Eeckloo. 2018. In defence of moral pluralism and compromise in health care networks. Health Care Analysis 26(4):362–79. 29594896 10.1007/s10728-018-0355-0 PMC6208978

[CIT0020] Savulescu, J . 2015. Bioethics: Why philosophy is essential for progress. Journal of Medical Ethics 41(1):28–33. 25516929 10.1136/medethics-2014-102284

[CIT0021] Scambler, G . 2001. Introduction: Unfolding themes of an incomplete project. In Habermas, Critical Theory, and Health, ed. G. Scambler, 1–24. London, United Kingdom: Routledge.

[CIT0022] Scambler, G., and N. Britten. 2001. System, lifeworld and doctor-patient interaction. In Habermas, Critical Theory, and Health, ed. G. Scambler, 52–4. London, United Kingdom: Routledge.

[CIT0023] Verlinden, A . 2010. Reconciling global duties with special responsibilities: Towards dialogical ethics. In Questioning Cosmopolitanism, eds. S. van Hooft and W. Vandekerckhove, 83–104. Dordrecht, Netherlands: Springer.

[CIT0024] Walker, P., and T. Lovat. 2016a. Dialogic consensus in clinical decision-making. Journal of Bioethical Inquiry 13(4):571–80. 27535798 10.1007/s11673-016-9743-z

[CIT0025] ———. 2016b. Towards a proportionist approach to moral decision making in medicine. Ethics and Medicine 32(3):153–61.

[CIT0026] ———. 2019. Dialogic consensus in medicine—A justification claim. The Journal of Medicine and Philosophy 44(1):71–84. 30590605 10.1093/jmp/jhy038

[CIT0027] Weinstock, D . 2013. On the possibility of principled moral compromise. Critical Review of International Social and Political Philosophy 16(4):537–56.

[CIT0028] ———. 2017. Compromise, pluralism and deliberation. Critical Review of International Social and Political Philosophy 20(5):636–55.

